# Characterization of the *Xylella fastidiosa* PD1671 Gene Encoding Degenerate c-di-GMP GGDEF/EAL Domains, and Its Role in the Development of Pierce’s Disease

**DOI:** 10.1371/journal.pone.0121851

**Published:** 2015-03-26

**Authors:** Luciana Cursino, Dusit Athinuwat, Kelly R. Patel, Cheryl D. Galvani, Paulo A. Zaini, Yaxin Li, Leonardo De La Fuente, Harvey C. Hoch, Thomas J. Burr, Patricia Mowery

**Affiliations:** 1 Department of Plant Pathology and Plant Microbe Biology, Cornell University, New York State Agricultural Experiment Station, Geneva, New York, United States of America; 2 Department of Biology, Hobart and William Smith Colleges Geneva, New York, United States of America; University of Zurich, SWITZERLAND

## Abstract

*Xylella fastidiosa* is an important phytopathogenic bacterium that causes many serious plant diseases including Pierce’s disease of grapevines. *X*. *fastidiosa* is thought to induce disease by colonizing and clogging xylem vessels through the formation of cell aggregates and bacterial biofilms. Here we examine the role in *X*. *fastidiosa* virulence of an uncharacterized gene, PD1671, annotated as a two-component response regulator with potential GGDEF and EAL domains. GGDEF domains are found in c-di-GMP diguanylate cyclases while EAL domains are found in phosphodiesterases, and these domains are for c-di-GMP production and turnover, respectively. Functional analysis of the PD1671 gene revealed that it affected multiple *X*. *fastidiosa* virulence-related phenotypes. A Tn5 PD1671 mutant had a hypervirulent phenotype in grapevines presumably due to enhanced expression of *gum* genes leading to increased exopolysaccharide levels that resulted in elevated biofilm formation. Interestingly, the PD1671 mutant also had decreased motility *in vitro* but did not show a reduced distribution in grapevines following inoculation. Given these responses, the putative PD1671 protein may be a negative regulator of *X*. *fastidiosa* virulence.

## Introduction

*Xylella fastidiosa* is a motile, xylem-limited bacterium transmitted to plants by xylem sap-feeding insects [1]. *X*. *fastidiosa* causes disease of many economically important crops, including Pierce’s disease in grapevines. It is proposed that once infection occurs, pathogenicity involves bacterial movement, aggregation, and occlusions of cells and biofilm formation, which leads to plugging and collapse of the xylem transport system [2].

*X*. *fastidiosa* moves against the transpiration stream using twitching motility [3], a flagella-independent movement involving the extension, attachment, and retraction of type IV pili [4]. *X*. *fastidiosa* translocation in plants requires degradation and movement through pit membranes [5,6], and plant primary cell walls are composed of celluloses, hemicelluloses, pectins, and proteins [7,8]. Once bacterial cells congregate, *X*. *fastidiosa* form biofilm, which is mediated by the type I pili [3] and non-fimbrial adhesins [9]. The biofilm is composed, in part, of extracellular polymeric substance (EPS) with fastidian gum, a derivative of xanthan gum, as the major constituent [10,11]. Fastidian gum production is regulated by the *gum* operon, of which *gumD* is postulated to initiate synthesis and *gumJ* plays a role in translocation of EPS [11,12]. Numerous molecular regulators are proposed to facilitate switching from motility to biofilm formation, such as the quorum sensing molecule diffusible signal factor (DSF) [13].

In many bacteria, movement and biofilm formation are inversely modulated by the intracellular second messenger, bis-(3’-5’)-cyclic dimeric guanosine monophosphate (c-di-GMP) [14]. C-di-GMP is synthesized by diguanylate cyclases (DGC) and hydrolyzed by phosphodiesterases (PDE); DGC catalyze two guanosine triphosphate (GTP) molecules to c-di-GMP using a GGDEF active site, and PDE degrade c-di-GMP to pGpG [5’-phosphogyanylyl-(3’-5’)-guanosine] using an EAL or HD-GYP domain. Hybrid proteins have been identified that contain both GGDEF and EAL domains [14–17].

*X*. *fastidiosa* has five putative proteins involved in production and turnover of c-di-GMP [17,18] three of which are characterized. CgsA has a GGDEF domain and is proposed to play a role in the transition of *X*. *fastidiosa* between vector and plant [13,19]. The Eal protein contains an EAL domain and was recently shown to be involved in antibiotic resistance and biofilm formation [20]. RpfG has an HD-GYP domain and is a response regulator involved in quorum sensing and biofilm formation [13]. The two uncharacterized proteins, PD1671 and PD1994, are annotated as containing both GGDEF and EAL domains [17]. We now report examination of the *X*. *fastidiosa* PD1671 protein, and provide evidence that its sequence is degenerative in both the GGDEF and EAL domains; however, it is involved in biofilm formation, presumably through regulation of *gum* gene expression and therefore EPS production, and subsequent Pierce’s disease development.

## Materials and Methods

### Bacterial strains

All studies involved *X*. *fastidiosa* strain Temecula 1, which was cultured at 28°C on modified periwinkle wilt (PW) agar [21] without phenol red and with 3.5 g L^-1^ bovine serum albumin (Life Technologies, Grand Island, NY). Mutants were cultured on modified PW containing 50 μg mL^-1^ kanamycin (Sigma Chemical Co., Saint Louis, MO). Complemented PD1671 strain was cultured on modified PW containing 50 μg mL^-1^ kanamycin and 5 μg mL^-1^ gentamycin (Sigma). Bacterial cultures were stored at -80°C on modified PW broth containing a final concentration of 7% DMSO (Sigma). *Escherichia coli* was cultured on Luria broth (LB) medium (Difco) with appropriate antibiotics (Sigma). Unless otherwise described, *X*. *fastidiosa*, mutants, and complemented mutant strains were cultured in spring-collected *Vitis vinifera* cv. Chardonnay xylem sap from California (kindly provided by Dr. A. Walker, University of California, Davis) for five days under agitation as previously described [22].

### Bioinformatic analysis

The predicted PD1671 gene product was characterized with BLAST searches on GenBank at National Center for Biotechnology Information together with PFAM [23], Conserved Domain Database [24], KEGG [25], and SMART programs [26]. Amino acids coding for various domains were determined using Conserved Domain Database [24]. ClustalW2 was used to generate multiple protein alignments [27,28]. Sequence comparisons for the different domains were made to relevant subsets of the following: *E*. *coli* CheY (WP_000763867), *Legionella pneumophila* Lpl0329 (WP_011214592), *Mycobacterium smegmatis* MSDGC-1 (WP_003893571), *Mycobacterium tuberculosis* Rv1354c (NP_215870), *Pseudomonas aeruginosa* FimX (WP_003123576), *Pseudomonas fluorescens* LapD (WP_011331847), *Pseudomonas putida* PP2258 (AE016418), *Rhodobacter sphaeroides* BphG1 (WP_011331450), *Vibrio parahaemolyticus* ScrC (WP_005478021), and *Xanthomonas oryzae* Flip (WP_012444707). Sequence comparisons for the different *X*. *fastidiosa* Temecula proteins were the following: CgsA (WP_011097571), RpfG (WP_011097637), EAL (WP_011098186), PD1671 (WP_011098203), and PD1994 (WP_004087568). Sequence comparisons of PD1671 to *X*. *fastidiosa* orthologs included the following: 9a5c strain (NP_297691), Ann-1 strain (AIC10147), Dixon strain (EAO14097), EB92 strain (EGO81612), M12 strain (YP_001776351), and M23 strain (YP_001830444).

### Identification of transposon insertion

We previously generated a transposon mutagenesis library (EZ-TN5^TM^ Epicentre, Madison, WI) of *X*. *fastidiosa* Temecula 1 [29]. Mutants were screened for impaired twitching motility on agar plates. Flanking regions of one such mutant, subsequently identified as the PD1671 deleted mutant, were amplified and cloned. DNA sequencing was performed at the Core Laboratories Center at Cornell University (Ithaca, NY). DNA sequences were submitted to the *X*. *fastidiosa* comparative genomic database [30] and submitted to BLAST program. Tn5 insertion was also confirmed by PCR (polymerase chain reaction) (Table 1).

**Table 1:** Oligonucleotide primers used in this study.

****Primers****	****Sequence 5’-3’****	****Use****	****Reference or source****
KAN-2 FP-1	ACCTACAAAGCTCTCATCAACC	Identify Tn insertion	Epicentre Biotech.
KAN-2 RP-1	GCAATGTA CATCAGAGATTTTGAG	Identify Tn insertion	Epicentre Biotech.
PD1671.F	GAATTCTTATTCAATTGGGGGTTACT	Complementation	This study
PD1671.R	GAATTCTCTTGTTTGAGTTTGCTATG	Complementation	This study
PD1671.RecF	CTTCAAGGTGCTGGCATACATG	RT-PCR^a^ PD1671 REC domain	This study
PD1671.RecR	GAAATCATCGGCACCACTATCA	RT-PCR PD1671 REC domain	This study
PD1671.GgF	TATGGTTACACTGCTTTCGAGC	RT-PCR PD1671 GGDEF domain	This study
PD1671.GgR	TTTAAAGCCTTGATTCAGCGGG	RT-PCR PD1671 GGDEF domain	This study
PD1671.EalF	CGTTCATCCACATAAACGTAGC	RT-PCR PD1671 EAL domain	This study
PD1671.EalR	GTTTGCAAGACGCAGTTATTCA	RT-PCR PD1671 EAL domain	This study
XP1275.RT.F	TTATGTAAGCGTCTTGGTGTGG	RT-PCR *dnaQ*	This study
XP1275.RT.R	GCACATGACCAGCGATCTTAC	RT-PCR *dnaQ*	This study
XP1455.RT.R	GGTGTGTGCATTTGCTTCTATG	RT-PCR *gumJ*	This study
XP1455.RT.F	GAGGAGAGTGAGGAAGGGATCT	RT-PCR *gumJ*	This study
XP1460.RT.R	TCTTCCGTGTCTTGGGATTC	RT-PCR *gumD*	This study
XP1460.RT.F	AATGACAGGCACATGACCAA	RT-PCR *gumD*	This study
RST31	GCGTTAATTTTCGAAGTGATTCGATTGC	Basipetal movement	[31]
RST33	CACCATTCGTATCCCGGTG	Basipetal movement	[31]

^a^ RT-PCR—reverse transcriptase-polymerase chain reaction.

### Genetic complementation

Total genomic DNA was extracted from 5 mL cultures of *X*. *fastidiosa* Temecula 1 using the Mo Bio Ultraclean Microbial DNA kit (Mo Bio Laboratories Inc., Carlsbad, CA). The PD1671 gene with 377 bases upstream for the promoter region was PCR amplified with primers PD1671F and PD1671R (Table 1). The resulting 2.4 kb product was digested with *EcoR*I and cloned into pBBR1MCS-5 [32], sequenced (Core Laboratories Center, Cornell University, Ithaca, NY), and electroporated into *X*. *fastidiosa* PD1671 mutant cells [29,33]. Cells were plated on PW agar amended with kanamycin and gentamycin. After 10 days colonies were screened for wild-type phenotypes.

### Bacterial growth and biofilm formation

Growth curves for wild-type, PD1671 mutant, and complemented PD1671 mutant were performed in *V*. *vinifera* xylem sap and biofilm formation was quantified with crystal violet staining as previously described [19,22]. Cells began at OD_600_ of 0.05 (1 x 10^6^ cells mL^-1^) for the growth curve and 0.1 (3 x 10^6^ cells mL^-1^) for biofilm assays. The growth curve was repeated five independent times with at least four replicates each time. Biofilm formation was measured in 96-well plates, and it was repeated with two independent experiments.

### Twitching motility in microfluidic chambers

The speed at which wild-type and mutant cells migrate against media flow was assessed in microfluidic devices as previously described [34]. Flow speed of Pierce’s Disease 2 medium (PD2) [35] controlled by a syringe pump was maintained at 1 x 10^4^ μm min^-1^. Cells attached to the glass surface of the microfluidic channels were observed microscopically using time-lapse image recordings every 30 s as previously reported [36]. Twenty five to thirty individual cells were tracked for the wild-type, PD1671 mutant, and complemented PD1671 mutant from three independent experiments. Data were analyzed with the Kruskal-Wallis test and means separated by the Kruskal-Wallis all pairwise comparison test.

### Exopolysaccharide (EPS) production assay

*X*. *fastidiosa* wild-type, PD1671 mutant, and complemented PD1671 mutant were grown in one mL *V*. *vinifera* Chardonnay xylem sap for five days at 28°C, with agitation (200 rpm). EPS production was quantified by dry weight [37]. Pearson’s Chi-square analysis was performed to compare EPS production by wild-type, PD1671 mutant, and complemented PD1671 mutant. The experiment was performed three independent times with five replicates each.

### Extracellular enzyme activity assays

The relative levels of carboxymethyl cellulase, endo-β-1,4-mannanase, polygalacturonase, and protease activity were assessed using radial diffusion assays, as described [37,38] with modifications. After cells were grown in one mL *V*. *vinifera* Chardonnay xylem sap for five days at 28°C, with agitation (200 rpm), and centrifuged, the supernatants were sterilized using filtration (0.2 μm). Three hundred microliters (6 x 50 μL) supernatant was then added to a 0.5 cm well that was created with a cork borer in the middle of a Petri plate. After 48 hr plates were stained (except protease assay) and enzyme activity was determined by measuring the zone diameter surrounding the supernatant cultures. Enzyme activity was visualized as clear or white halos surrounding the wells. Pearson’s Chi-square analysis was performed to compare the results. Assays were performed three times with five replicate each.

For the carboxymethyl cellulose assay, plates containing medium was used containing 0.1% carboxymethyl cellulose, 25 mM sodium phosphate, pH 7.0, and 0.8% agarose [37]. Plates were stained with 0.1% Congo red for 20 min and then were washed twice with 1 M NaCl. For the endo-β-1,4-mannanase assay, media was prepared by with 0.5 g locust bean gum galactomannan dissolved in 500 mL of McIlvaine buffer (pH 5) [37,38]. The suspension was heated to 80°C while constantly stirring for 2 hr, removed from heat, and stirred continually overnight. The suspension was centrifuged at 5,000g. Gelatin was added to the mix (250 mg L^-1^) and sterilized by autoclave. The plate was washed for 30 min in a 1.0:4.3 of 0.1 M citric acid and 0.2 M Na_2_HPO_4_ (pH 7.0), stained for 30 min in 0.5% (w/v) Congo red dye, washed in water for 2 min, fixed with 80% (v/v) ethanol for 10 min, washed in water again for 2 min, washed with three 20 min washes in McIlvaine buffer (pH 7), and developed after five washes with 1 M NaCl. To test for polygalacturonase activity, a medium was used containing 1.0% agarose and 0.1% polygalacturonic acid was dissolved in 100 mM potassium phosphate (pH 6.5) [37]. Surface of the medium was covered with 0.05% (w/v) ruthenium red for 20 min and rinsed 5 times in water. For the protease assay, activity was determined using PW plates containing 0.5% skimmed milk [37].

### Semiquantitative RT-PCR (reverse transcriptase-PCR) analysis

Bacterial cultures were grown for four days in *V*. *vinifera* xylem sap to approximately 10^7^ cell mL^-1^ (OD_600_≈1.0) and then harvested by centrifugation at 16,000g at 4°C for 5 minutes. The cells were suspended in RNeasy lysis buffer (Qiagen, Valencia, CA), and then lysed by Trizol reagent (Invitrogen, Carlsbad, CA). RNA was purified using RNeasy columns (Qiagen) and treated with DNase I (Invitrogen). RNA was quantified using a Nanodrop 1000 spectrophotometer (NanoDrop, Wilmington, DE) and qualitatively analyzed on agarose gels, then 5 μg of total RNA was used for cDNA synthesis using the first-strand cDNA using SuperScript III One-Step RT-PCR System with Platinum Taq polymerase according to the manufacturer’s protocol (Invitrogen). The resulting cDNA was utilized for regular PCR with gene or domain-specific primers (Table 1). Aliquots of each amplicon were electrophoresed on a 1.2% agarose gel and visualized by ethidium bromide staining. Gels were photographed using the Kodak Image Station 440CF (Eastman Kodak Company, Rochester, NY) and images densitometrically quantified with the Image J software (National Institutes of Health, Bethesda, MD) according to the manufacturer’s instructions. The experiment was performed three to six independent times with three replicates each.

### Virulence assays in grapevines

The pathogenicity assay was performed [3,39,40] with modifications. A 20 μL aliquot of an OD_600_ = 2.0 (about 8 x 10^9^ cells mL^-1^) suspension of wild-type and PD1671 mutant cells, cultured in *V*. *vinifera* xylem sap for four days or from PW plates, was inoculated individually by a needle puncture method (BD ultrafine short insulin pen needle, BD Biosciences, Franklin Lakes, NJ) into grapevines (*V*. *vinifera* L. cv. Cabernet Sauvignon) that had been grown for two months in the greenhouse. The cell suspension was inoculated into the stem at a position seven internodes up from the base of the shoot (ten plants per treatment). The negative control consisted of plants inoculated with succinate-citrate buffer [41]. Plants were scored weekly (until week twenty) for disease symptoms on a scale from zero to five [9]. The experiment was repeated three independent times and data were analyzed by repeated measures ANOVA.

### Basipetal movement in plants

The basipetal movement of wild-type and PD1671 mutant in inoculated plants was performed as previously described [3] with modifications. Two month old *V*. *vinifera* L. cv. Cabernet Sauvignon plants varying from 60–100cm in height were inoculated by needle puncture, as described above. At least four shoots were examined after four, eight, and twelve weeks and the bacterial population determined by agar plate count. Shoots were cut from the main trunk and surface sterilized (70% ethanol for one min, 2% sodium hypochlorite solution for two min, and 70% ethanol for 30 s, followed by two rinses in sterile water). Subsequently, one cm sections were aseptically excised at measured distances from the original point of inoculation. The one cm sections were crushed in sterile polycarbonate bags containing 200 μL of sterile succinate-citrate buffer. The liquid phase resulting from the triturate was spread onto modified PW agar (wild-type *X*. *fastidiosa*) or PW agar containing kanamycin (PD1671 mutant). The plates were examined for the presence of *X*. *fastidiosa* after seven days at 28°C by measuring colony forming units (CFU) and PCR. PCR was performed using *X*. *fastidiosa* specific primer pairs RST31/RST33 (Table 1).

### Statistical analysis

Statistical analyses were performed using the statistical software package SPSS version 18.0 or 19.0 (SPSS Inc., Chicago, IL). All quantitative assays, unless otherwise specified, were analyzed using one-way analysis of variance.

## Results

### Identifying a PD1671 mutant

A twitching-defective Tn5 mutant was identified with a disruption in open reading frame PD1671 at codon 468 of 566; PD1671 is predicted to encode a 566 amino acid protein. The Tn5 insertion abolished transcription from the gene segment examined, as determined by RT-PCR using *dnaQ* (PD1217) as an internal control (Table 2). The REC and GGDEF domains of PD1671 retained wild-type level expression. Whether the REC-GGDEF mRNA translated into functional and stable protein was not measured, however, we can assume PD1671 mutant phenotype findings reflected the PD1671 gene with a loss of the EAL domain.

**Table 2:** Relative *X*. *fastidiosa* RNA levels.

****Gene or domain**** ^a^	****Wild-type****	****PD1671 mutant****	****Complemented PD1671****
PD1671 REC domain	1.11 ± 0.11	0.99 ± 0.07	1.78 ± 0.62
PD1671 GGDEF domain	0.96 ± 0.06	0.92 ± 0.18	1.20 ± 0.30
PD1671 EAL domain	0.96 ± 0.08	0.00 ± 0.00^b^	1.40 ± 0.62
*gumD*	1.18 ± 0.45	2.86 ± 0.31^b^	1.02 ± 0.01
*gumJ*	1.36 ± 0.33	2.61 ± 0.45^b^	1.25 ± 0.22

^a^ RT-PCR (reverse transcriptase-polymerase chain reaction) experiments performed in *Vitis vinifera* xylem sap (three to six independent experiments with three replicates each). The standard deviations of the normalized means are shown. Expression of the gene regions was normalized to *dnaQ* gene expression [42]. Gene segments amplified: PD1671-REC domain (115 to 357bp), PD1671-GGDEF domain (601 to 858bp), PD1671-EAL domain (1325 to 1621bp), *gumD* (628–847bp), and *gumJ* (4–229bp).

^b^ Statistically significant compared to wild-type (*P*<0.01).

**Fig 1:**
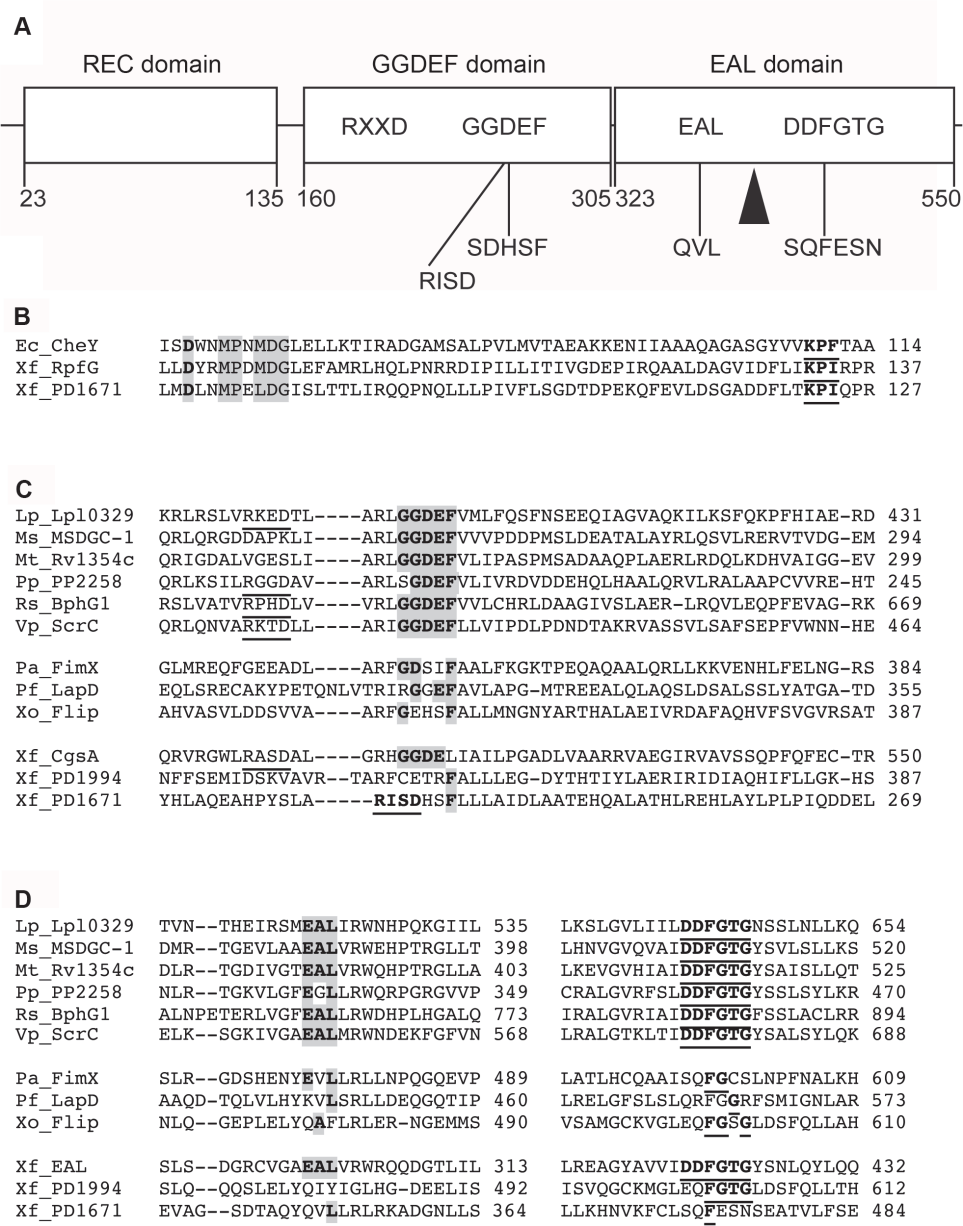
Putative PD1671 domains. **A**) Boxes represent the three PD1671 domains with domain names above the boxes and amino acid numbers below the boxes. Bacterial diguanylate cyclase and phosphodiesterase consensus sequences listed in boxes (X is any amino acid), and PD1671 aligned sequences listed below the boxes at their approximate locations. Arrow head denotes Tn5 insertion point. **B**) REC domain alignment. *Xylella fastidiosa* PD1671 REC domain alignment with functional REC protein and *X*. *fastidiosa* predicted c-di-GMP protein containing REC domain. Grey boxed/bold amino acids are the phosphorylation site, grey boxed/non-bold amino acids are the intermolecular recognition site, and bold/underlined amino acids are the dimerization interface. **C**) GGDEF domain. Top sequence group is hybrid GGDEF-EAL domain-containing proteins enzymatic in both domains, middle sequence group is non-enzymatic hybrid GGDEF-EAL domain-containing proteins, and bottom sequence group is *X*. *fastidiosa* predicted GGDEF domain proteins. Underlined amino acids are the allosteric I site, RxxD, and grey boxed/bold amino acids are the GGDEF sequences. Underlined/bold PD1671 residues denote a potential RxxD site. **D**) EAL alignment. Top sequence group is hybrid GGDEF-EAL containing proteins enzymatic in both subunits, middle sequence group is non-enzymatic hybrid GGDEF-EAL domain proteins, and bottom sequence group is *X*. *fastidiosa* predicted EAL proteins. Grey boxed/bold amino acids are signature EAL sequence and underlined/bold residues are DDFGTG sequences. Alignment comparison sequences: Ec = *Escherichia coli*, Lp = *Legionella pneumophilia*, Ms = *Mycobacterium smegmatis*, Mt = *Mycobacterium tuberculosis*, Pa = *Pseudomonas aeruginosa*, Pf = *Pseudomonas fluorescens*, Pp = *Pseudomonas putida*, Rs = *Rhodobacter sphaeroides*, Vp = *Vibrio parahaemolyticus*, Xf = *Xylella fastidiosa*, Xo = *Xanthomonas oryzae*.

We examined the three major domains of PD1671: i) an N-terminal REC (response regulator, signal receiver, CheY-like receiver) domain, ii) a GGDEF domain, and iii) an EAL domain (Fig. 1A). i) We compared the PD1671 REC domain to the well-characterized *E*. *coli* CheY REC protein [43] and the relevant domain of *X*. *fastidiosa* REC-containing c-di-GMP protein RpfG [17] (Fig. 1B). The PD1671 REC domain has a predicted phosphorylation site, Mg^2+^ active site, and a dimerization interface. For the GGDEF and EAL domain comparisons, we aligned PD1671 with hybrid GGDEF-EAL domain-containing proteins with known enzymatic activity in both subunits [44–49], hybrid non-enzymatic GGDEF-EAL domain-containing proteins with alternative c-di-GMP function [50–52], and the four predicted *Xylella fastidiosa* DGC and/or PDE proteins [17]. ii) The putative Temecula 1 PD1671 GGDEF domain appears to lack the signature motif (Fig. 1C). iii) For the predicted *X*. *fastidiosa* PD1671 EAL domain, the conserved DDFGTG segment appears absent (Fig. 1D). For each of the three PD1671 domains, the critical conserved sequences described above for *X*. *fastidiosa* Temecula 1 are conserved across *X*. *fastidiosa* strains 9a5c, Ann-1, Dixon, EB92, M12, and M23 (S1 Fig).

### Growth and motility

**Fig 2:**
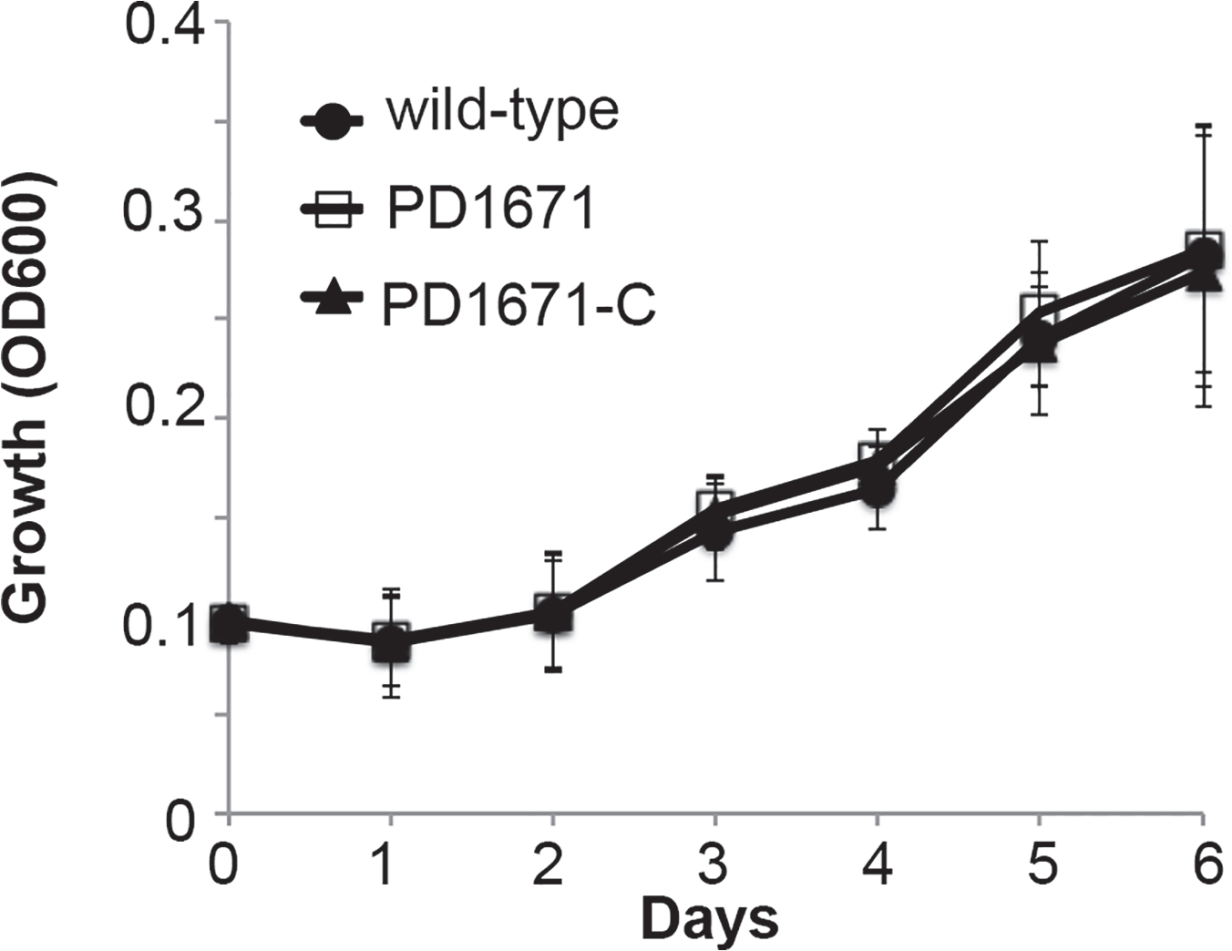
Growth curve of the *X*. *fastidiosa* PD1671 mutant. Growth of wild-type *X*. *fastidiosa* Temecula 1 (circle), PD1671 mutant (square), and complemented PD1671 mutant (PD1671-C) (triangle) strains in *Vitis vinifera* Chardonnay xylem sap. Cell densities were measured daily at OD_600_. Average and standard deviation of the averages of five independent experiments with a minimum of four replicates each (*P* = 0.927).

**Fig 3:**
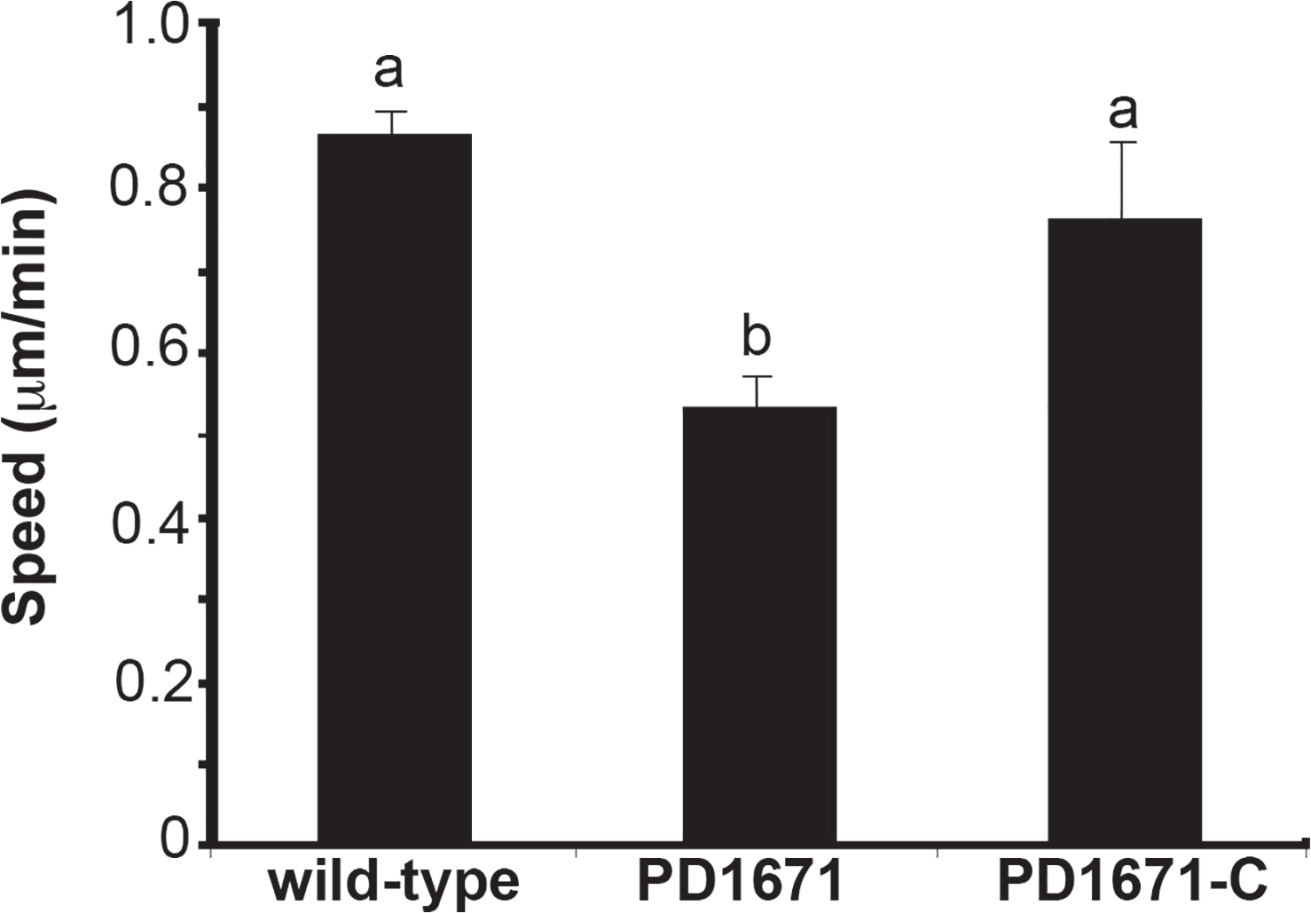
Movement of the *X*. *fastidiosa* PD1671 mutant in microfluidic chambers. Twitching movement speed of wild-type *X*. *fastidiosa* Temecula 1, PD1671 mutant, and complemented PD1671 mutant (PD1671-C) cells in microfluidic flow chambers. Values shown are means and standard errors from three independent experiments. Letters above bars indicate significant differences by Kruskal-Wallis test and means were separated by the Kruskal-Wallis all pairwise comparison test (*P* = 0.02)

The PD1671 mutant, complemented PD1671 mutant, and wild-type cells grew equally in *V*. *vinifera* xylem sap (*P* = 0.927) (Fig. 2). Concerning motility, we examined the speed of strains under flow conditions in microfluidic chambers and found that the PD1671 mutant had reduced motility compared to wild-type or complemented mutant cells (*P* = 0.02) (Fig. 3).

### Expression of *gum* genes and extracellular enzyme activity

The *gumD* and *gumJ* genes in the mutant strain were upregulated approximately two-fold to that of the PD1671 complemented mutant and wild-type cells (*P*<0.01) (Table 2). By our assays, the PD1671 mutant strain had no effect on carboxymethyl cellulase (endo-1,4-β-glucanase) or undefined protease function, while endo-β-1,4-mannanase (a hemicellulase) and polygalacturonase (pectin-degrading enzyme) activity was not detected for any strains tested (Table 3).

**Table 3:** Relative *X*. *fastidiosa* exoenzyme activity.

****Extracellular enzyme**** ^a^	****Wild-type****	****PD1671 mutant****	****Complemented PD1671****
Carboxymethyl cellulase	1.01 ± 0.20	1.10 ± 0.30	1.20 ± 0.25
Endo-β-1,4-mannanase	0.00 ± 0.00	0.00 ± 0.00	0.00 ± 0.00
Polygalacturonase	0.00 ± 0.00	0.00 ± 0.00	0.00 ± 0.00
Protease	2.12 ± 0.20	2.20 ± 0.30	2.10 ± 0.25

^a^ Extracellular enzyme activities were estimated from the diameter (mm) of the halo zones of supernatant enzymatic activity surrounding each well. All assays were performed three times, with five replicate plates each. The standard deviations of the means for each enzyme are shown.

### EPS production and biofilm formation

**Fig 4:**
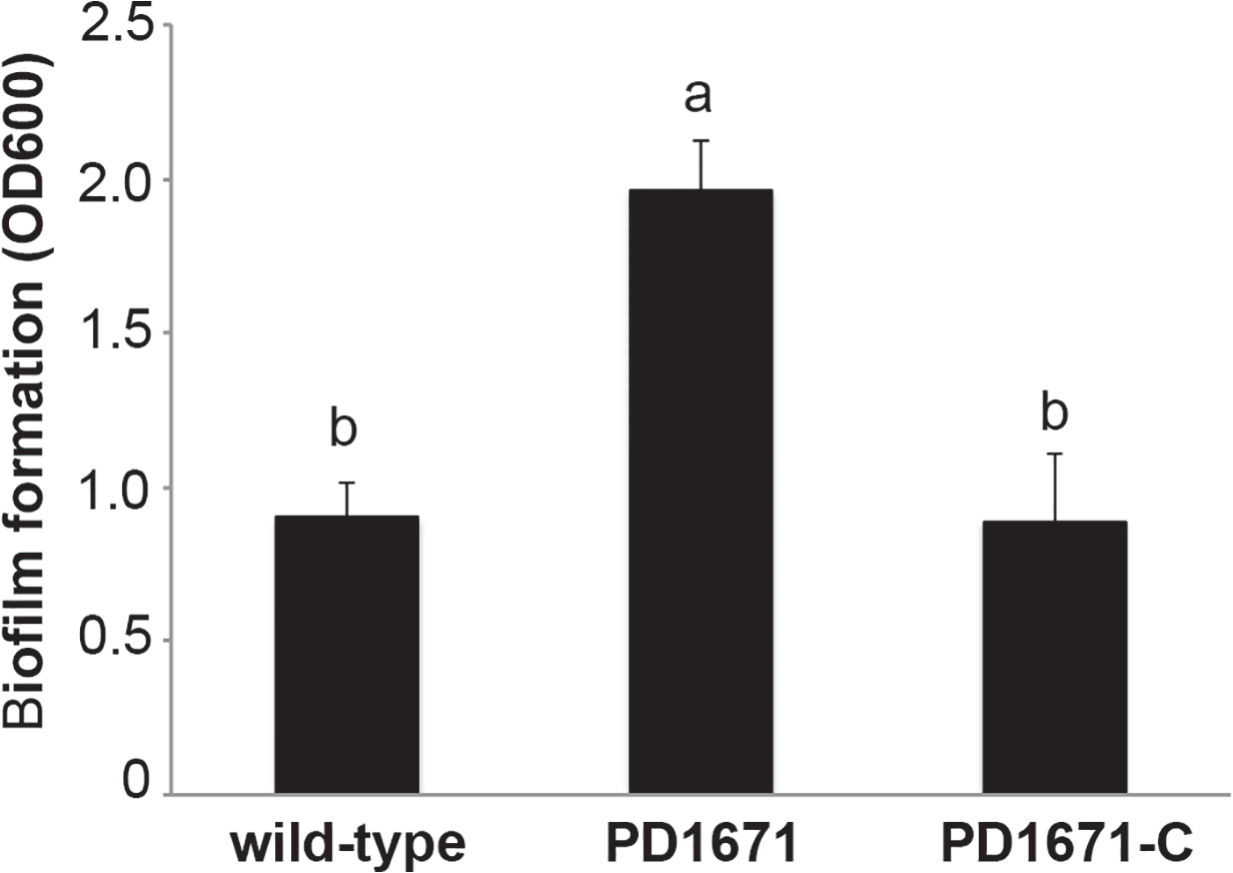
Biofilm production by the *X*. *fastidiosa* PD1671 mutant. Quantification of biofilm formation in *Vitis vinifera* xylem sap with agitation for wild-type *X*. *fastidiosa* Temecula 1, PD1671 mutant, and complemented PD1671 mutant (PD1671-C). Representative experiment shown. Different letters represent significant difference when means are compared (*P* = 0.001).

The amount of EPS was over two times greater in the PD1671 mutant in comparison to wild-type cells (*P*<0.0001) (Table 4). When biofilm formation was examined [19,22], we found that the PD1671 mutant formed more biofilm than wild-type cells (*P* = 0.001) (Fig. 4). For EPS production and biofilm formation assays the complement strain restored the phenotypes to those observed with the wild-type strain.

**Table 4:** Relative *X*. *fastidiosa* exopolysaccharide (EPS) production.

	****Wild-type****	****PD1671 mutant****	****Complemented PD1671****
**EPS production** ^a^	1.11 ± 0.03	2.49 ± 0.43^b^	1.11 ± 0.02

^a^ Average dry weight of EPS (mg mL^-1^). The experiments were performed three times with five replicates each. The standard deviations are shown.

^b^ Statistically significant compared to wild-type (*P*<0.0001).

### PD1671 mutant *in planta*

**Fig 5:**
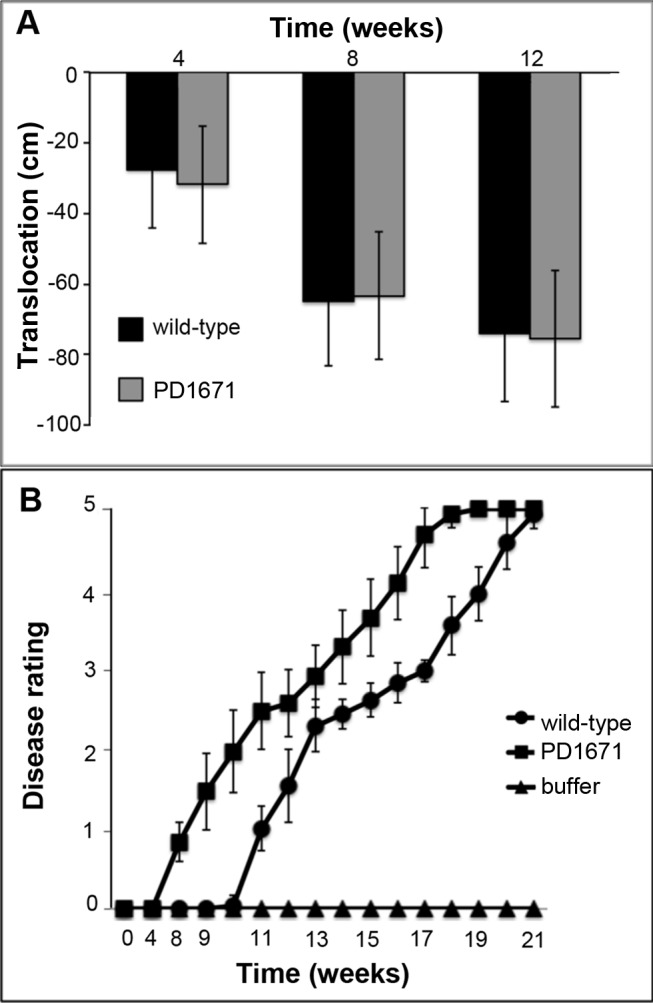
Movement and virulence in grapevines by the *X*. *fastidiosa* PD1671 mutant. **A**) Basipetal translocation *in planta* of PD1671 mutant compared to wild-type *X*. *fastidiosa* Temecula 1. Average distances at which cells were recovered from vine regions upstream of the inoculation point (represented by 0 on the y axis) after four, eight, and twelve weeks post-inoculation. Error bars represent standard deviation of the means of at least four plants (*P* = 0.40). **B**) Severity of Pierce's disease. Average of at least 10 plants per treatment with Temecula 1 (circle), PD1671 mutant (square), and buffer negative control buffer (triangle). Experiment was repeated three times. Error bars represent standard deviation of the means of a representative experiment (*P*<0.005).

*V*. *vinifera* vines inoculated with the mutant and wild-type *X*. *fastidiosa* cells were examined for disease progression. When examined over time, both wild-type and PD1671 mutant strains were recovered from grapevine shoot sections upstream of the inoculation site with no significant differences in numbers (*P* = 0.40) (Fig. 5A). After twelve weeks both bacterial strains had completely translocated from the point of inoculation to the end of the shoots with comparable concentrations (1x10^8^ CFU mL^-1^ for PD1671 mutant and 1.3x10^8^ CFU mL^-1^ for wild-type). Plants inoculated with the PD1671 mutant and wild-type cells induced a similar overall pattern of disease progression, however, the mutant-induced disease was shifted; PD1671 mutant-inoculated plants showed disease symptoms three weeks earlier (*P*<0.005) and progressed to full disease rating three weeks sooner than plants inoculated with wild-type cells (Fig. 5B).

## Discussion

In this report we demonstrate that a putative *X*. *fastidiosa* PD1671 protein is involved in *gum* gene expression, EPS production, biofilm formation, and Pierce’s disease development. The PD1671 gene is annotated as belonging to a two-component regulatory system [18], and is considered an orphan response regulator [53]. It contains an *N*-terminal REC domain of unknown function. Throughout bacterial genomes, REC domains are found as stand-alone proteins or as domains within larger proteins [54]. In GGDEF and EAL domain-containing proteins, phosphorylation of the REC domain is hypothesized to induce dimerization and function [15]. Given that the putative PD1671 REC domain has a predicted dimerization domain and the conserved aspartic residue for phosphorylation that aligns with the *E*. *coli* Asp57 [24,55,56], the domain may be functional.

Previous work identified the *X*. *fastidiosa* PD1671 as a hybrid GGDEF-EAL domain-containing protein [17]. When compared to known enzymatic GGDEF-EAL proteins [44–49], we found that the predicted PD1671 protein is lacking or degenerative in critical binding and active sites in both domains. Of note, the PD1671 EAL sequence is degenerate, however, a valine replacing alanine is the most common substitution in this motif [14]. GGDEF-EAL hybrid domain proteins are frequently inactive in one or both domains [15–17]. Those with degenerate sequences are found to have biological functions that appear to be either c-di-GMP dependent or independent [50–52,57]. PD1671 more closely aligns in the key conserved sequences with known non-enzymatic, c-di-GMP binding hybrid GGDEF-EAL proteins FimX, Flip, and LadD. Non-enzymatic GGDEF-EAL proteins can function as c-di-GMP receptors [58] by binding the ligand in either the EAL-like domain at an unknown position or in the GGDEF domain at the allosteric I RxxD site [51,59]. However, while the putative PD1671 has an RxxD sequence near the conserved position, it lacks the expected five residue linker between the RxxD sequence and the aligned GGDEF sequence [60] so it may not be functional.

### Regulation of biofilm formation

*X*. *fastidiosa* forms biofilm that, along with increased bacterial concentration, is proposed to clog xylem vessels leading to disease symptoms [2]. *X*. *fastidiosa* produces EPS, which has been shown to be an important physical component of biofilm [61]. Based on homology to *Xanthomonas* species, the *X*. *fastidiosa* GumD protein is proposed to be part of a EPS polysaccharide assembly, and the GumJ protein is involved in EPS secretion [61–64]. Our results suggest that the uninterrupted putative PD1671 protein downregulates *gum* gene expression, which presumably limits EPS production and therefore reduces biofilm formation. Thus, when the PD1671 protein is fully expressed, it appears to be an anti-virulence regulator. In support of this, disruption of the *X*. *fastidiosa gumD* gene correlates with reduced EPS and biofilm production and an avirulent phenotype in grapevines [61].

### Role in motility and exoenzyme activity

In microfluidic chambers, the PD1671 mutant migrated slower than wild-type cells. Reduced motility may suggest that in wild-type cells the PD1671 EAL domain may have a direct regulatory role in motility, and c-di-GMP is known to be important in type IV pili regulation and response [14]. Alternatively, the observed reduced motility may result from increased EPS production and biofilm formation that impedes movement. However, a difference in motility was not detected between the PD1671 mutant and wild-type cells *in planta*. The *in vivo* and *in vitro* environments may be distinct enough to produce different observed responses. For instance, results may reflect the difference in assay timeframes or that each environment induced unique gene expression profiles that altered findings [42,65–68].

Translocation requires degradation of pit membranes and two *X*. *fastidiosa* exoenzymes have been studied, polygalacturonase and endo-1,4-β-glucanase [69,70]. In our assays, these and the other the extracellular enzymes tested showed no differences in the PD1671 mutant compared to wild-type or complemented strains. Of note, polygalacturonase activity in media or xylem fluid has been reported to be unsuccessful or inconsistent [69,71]. Overall our findings suggest that the predicted PD1671 protein does not have an *in vivo* regulatory role in *X*. *fastidiosa* twitching motility or movement through pit membranes.

### Effects on virulence

Motility and biofilm formation are thought to be key in *X*. *fastidiosa*-induced disease. Motility has been found to have a direct relationship with disease. *X*. *fastidiosa* mutants exhibiting increased motility are hypervirulent [9], while those with decreased motility have reduced pathogenicity [39,72]. While our PD1671 mutant strain was hypervirulent, it exhibited equal movement to wild-type cells *in planta*, suggesting that other factors explain the phenotype. Biofilm, composed in part by EPS, also has been found to have a direct relationship with symptom development. *X*. *fastidiosa* mutants with decreased biofilm have decreased virulence [39,61,72–74], suggesting that mutants with increased biofilm formation might be hypervirulent. The PD1671 mutant showed increased biofilm production and so might be expected to have increased pathogenicity. Therefore, we suspect that the hypervirulent phenotype of the PD1671 mutant resulted from upregulated *gum* gene expression that leads to increased EPS, which in turn increased biofilm production.

### PD1671 involvement in regulating multiple responses

The observed phenotypes, along with degenerated protein sequences, suggest that the putative PD1671 plays a regulatory role, as other non-catalytic GGDEF-EAL hybrid domain-containing proteins have been implicated in regulation processes [57,58]. Motility and biofilm formation are regulated by a number of genes in *X*. *fastidiosa*. We previously identified a chemotaxis homologous operon, Pil-Chp, which regulates twitching motility [72]. In addition, the *pilR*/*pilS* two-component regulatory system regulates the transcription of *pilA*, the major pilin of the type IV pilus that controls twitching motility [29,75]. Biofilm formation and the pili genes are associated with sigma factor 54 (RpoN) [76] and the global regulators *algU* and *gacA* [73,77]. DSF and c-di-GMP have been shown to be involved in regulating biofilm and motility. High levels of DSF in *X*. *fastidiosa* are proposed to correlate with reduced motility, upregulated *gum* and adhesion genes, increased EPS production, and enhanced aggregation of cells with biofilm potentially by signaling through RpfG and altering c-di-GMP levels [13].

Given how enzymatically active GGDEF and EAL domain-containing proteins function, mutations in PDE proteins would be expected to show decreased motility and increased *gum* gene expression, EPS production, and biofilm formation, while mutations in DGC proteins would be predicted to exhibit the opposite phenotypes. While we cannot rule out a potential dominant-negative effect from the transposon insertion in the 3’ end of PD1671 [78–80], the phenotypes of our PD1671 mutant matches the predicted responses of the protein lacking an EAL domain. Therefore the observed responses of the PD1671 mutant suggest it might be directly or indirectly involved in decreasing c-di-GMP levels. However, the *X*. *fastidiosa* c-di-GMP proteins that have been directly examined exhibit unexpected phenotypes. Deletion of the *X*. *fastidiosa eal* PDE-encoding gene leads to decreased *gum* gene expression, EPS production, and biofilm formation and increased motility [20]. Mutation of the *X*. *fastidiosa csgA* DGC-encoding gene showed increased *gum* gene expression, EPS production, and biofilm formation but decreased virulence [19]. Whether the predicted PD1671 is involved in c-di-GMP levels and whether it associates with other *X*. *fastidiosa* regulatory systems are still areas of exploration. Understanding how the putative PD1671 regulates pathogenic responses will provide greater insight into the signaling mechanisms controlling biofilm-forming behaviors critical for Pierce’s disease development.

## Supporting Information

S1 Fig Putative PD1671 domains aligned with ortholog *X*. *fastidiosa* proteins. Stars represent amino acids that are not conserved across the orthologs. **A**) REC domain alignment. Grey boxed/bold amino acids are the phosphorylation site, grey boxed/non-bold amino acids are the intermolecular recognition site, and bold/underlined amino acids are the dimerization interface. **B**) GGDEF alignment. Underlined/bold PD1671 residues note a potential RxxD site. Grey boxed/bold amino acids are the conserved residues matching the GGDEF sequence as seen in Fig. 1C. **C**) EAL alignment. Grey/boxed residues match the signature EAL sequence and the underlined/bold residues line with the DDFGTG sequences as seen in Fig. 1D.
(TIF)
