# Supplementary figures and images for: Characterization of the Xylella fastidiosa PD1671 Gene Encoding Degenerate c-di-GMP GGDEF/EAL Domains, and Its Role in the Development of Pierce’s Disease
Source: PLoS One. 2015 Mar 26;10(3):e0121851. doi: 10.1371/journal.pone.0121851 (PMC4374697; doi:10.1371/journal.pone.0121851)

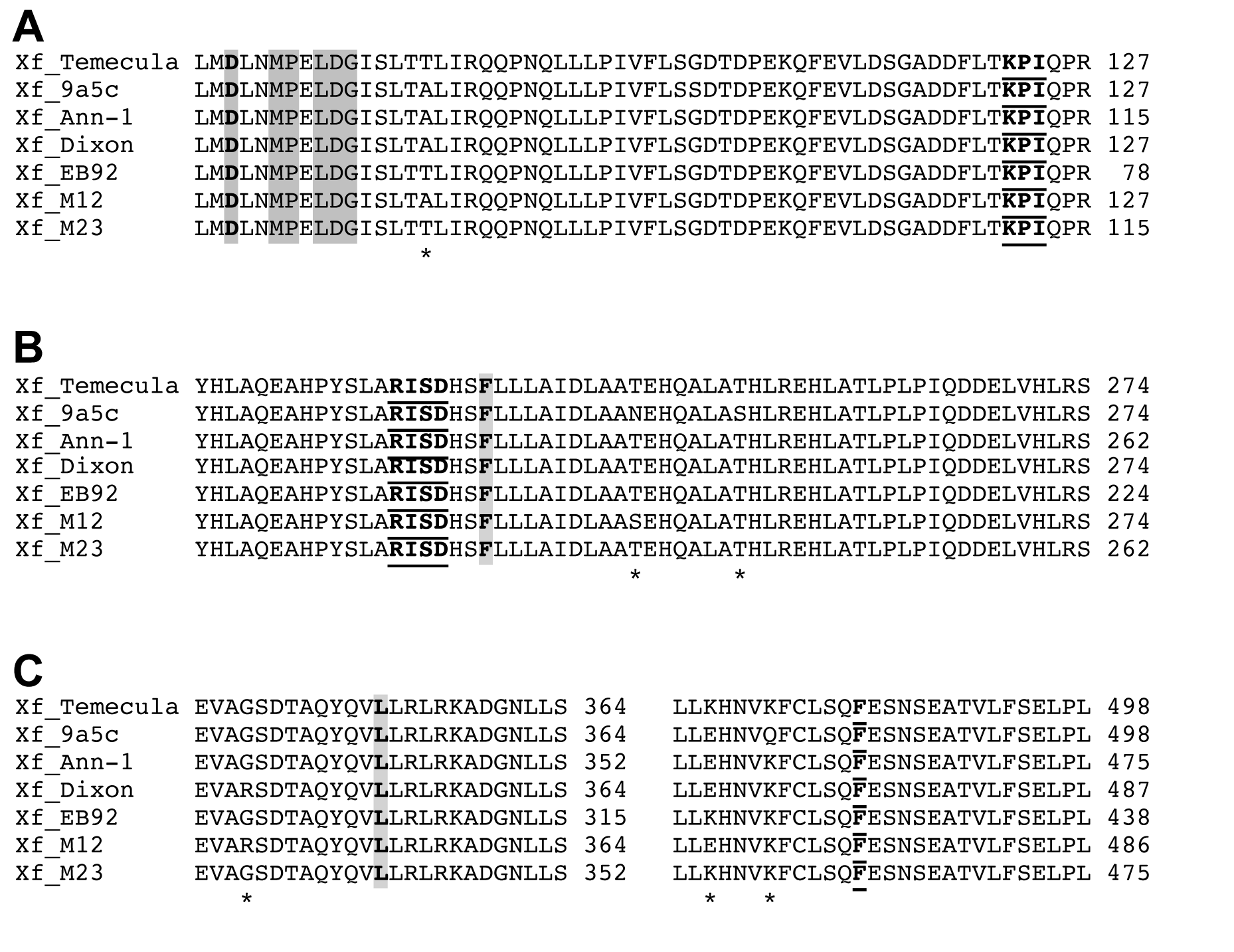

Supplement: S1 Fig — Stars represent amino acids that are not conserved across the orthologs. A) REC domain alignment. Grey boxed/bold amino acids are the phosphorylation site, grey boxed/non-bold amino acids are the intermolecular recognition site, and bold/underlined amino acids are the dimerization interface. B) GGDEF alignment. Underlined/bold PD1671 residues note a potential RxxD site. Grey boxed/bold amino acids are the conserved residues matching the GGDEF sequence as seen in Fig. 1C. C) EAL alignment. Grey/boxed residues match the signature EAL sequence and the underlined/bold residues line with the DDFGTG sequences as seen in Fig. 1D. (TIF) [file pone.0121851.s001.tif]
